# Comparison of bacterial profile of gallbladder with gallstones from patients undergoing cholecystectomy due to complicated and uncomplicated cholelithiasis: changes in the epidemiological scenario

**DOI:** 10.1590/0100-6991e-20233474-en

**Published:** 2023-04-12

**Authors:** BRUNO HENRIQUE NUNES HIRATA, SUZETHE SASAGAWA, ALESSANDRA NAVARINI, HENRIQUE CUNHA MATEUS, ADHEMAR MONTEIRO PACHECO, MAURO JOSÉ COSTA SALLES

**Affiliations:** 1 - Faculdade de Ciências Médicas da Santa Casa de São Paulo - São Paulo - SP - Brasil; 2 - Faculdade de Ciências Médicas da Santa Casa de São Paulo, Departamento de Microbiologia - São Paulo - SP - Brasil; 3 - Hospital de Misericórdia da Santa Casa de São Paulo, Cirurgia - São Paulo - SP - Brasil; 4 - Hospital de Misericórdia da Santa Casa de São Paulo, Infectologia - São Paulo - SP - Brasil

**Keywords:** Microbiology, Cholecystectomy, Cholecystitis, Acute, Cholelithiasis, Microbiologia, Colecistite Aguda, Colelitíase, Colecistectomia

## Abstract

**Introduction:**

cholelithiasis is a highly prevalent disease of the digestive system in the world. In Brazil, it is a routine condition, whose studies suggest a prevalence of around 10% of adults. Colonization of bile and gallstone pathogens can occur when there is bacterial stasis and proliferation. This proliferation is facilitated by the adhesion and biofilm formation capacity of some bacteria. There are also lithogenic processes that involve bacterial participation. Studies have shown changes in the microbiota of the gallbladder of patients undergoing cholecystectomy, which may impact empirical treatment with antibiotics.

**Methodology:**

microbiological analyzes of the sonication fluid of the gallstones and of two samples with bile were performed. Identification and antimicrobial susceptibility testing were performed according to a standard routine.

**Results:**

of the 34 patients, 76.4% were female. The age group was 48 years +/- 16.61. Acute cholecystitis occurred in 50% of cases. Bactobilia was evidenced in 32.1% of the cases. Klebisiella pneumoniae was noted as the most prevalent pathogen in acute cholecystitis; and Enterobacter sp, in cases of uncomplicated cholelithiasis. Greater sensitivity was obtained in the search for microorganisms in the sonication fluid samples of the stones in relation to the bile samples (p=0.0058).

**Conclusion:**

there was a higher prevalence of bactobilia in patients with acute cholecystitis compared to those with uncomplicated cholelithiasis. The use of sonication in bacterial investigation proved to be superior to the conventional method and can be considered.

## RESUMO

**Introdução:**

a colelitíase é uma doença do sistema digestivo de alta prevalência no mundo. No Brasil, trata-se de uma condição rotineira, cujos estudos sugerem uma prevalência em torno de 10% dos adultos. A colonização de patógenos da bile e do cálculo biliar pode ocorrer quando há estase e proliferação bacteriana. Esta proliferação é facilitada pela capacidade de adesão e de formação de biofilme de algumas bactérias. Também há processos litogênicos que envolvem a participação bacteriana. Estudos evidenciaram mudanças na microbiota da vesícula biliar de pacientes submetidos à colecistectomia, o que pode impactar no tratamento empírico com antibióticos.

**Metodologia:**

Realizou-se análises microbiológicas do fluido de sonicação dos cálculos biliares e de duas amostras com bile. A identificação e o teste de sensibilidade aos antimicrobianos foram realizados de acordo com uma rotina padrão.

**Resultados:**

Nos 34 pacientes, 76,4% deles foram do sexo feminino. A faixa etária foi de 48 anos +/- 16,61. A colecistite aguda ocorreu em 50% dos casos. A bactobilia foi evidenciada em 32,1% dos casos. Notou-se Klebisiella pneumoniae como o patógeno mais prevalente em quadros de colecistite aguda; e Enterobacter sp, nos casos de colelitíase não complicada. Obteve-se maior sensibilidade na pesquisa de microrganismos nas amostras de fluido de sonicação dos cálculos em relação às de bile (p=0,0058).

**Conclusão:**

Houve maior prevalência de bactobilia nos pacientes com colecistite aguda em relação àqueles com colelitíase não complicada. A utilização da sonicação na investigação bacteriana se mostrou superior ao método convencional e pode ser considerada.

## INTRODUCTION

Cholelithiasis is a highly prevalent disease^1^ ^,^ ^2^. Approximately 10% of Brazilians have gallstones^3^ ^,^ ^4^. Bile acquires lithogenic capacity when there is a change in its content. The majority (>80%) of gallstones are of cholesterol, yellow in color^1^ ^,^ ^7^. The formation of pigmented calculi is the result of cholestasis associated with bacterial colonization in the biliary tract^5^ ^,^ ^6^ and/or biliary parasites^7^ ^-^ ^8^. Although cholelithiasis is a predominantly asymptomatic disease, gallstones are associated with important complications, such as acute cholecystitis (AC)^9^.

Assessment of microbial profiles and antibiotic sensitivity in bile cultures can help guide antibiotic therapy^10^ ^-^ ^12^. Jijun Zhao et al. observed that, of 789 bile cultures, 574 were positive (73%). Of these, 386 were gram-negative bacilli (67%), 170 were gram-positive cocci (30%), and 18 were fungi (3%). The most common organisms were enteric bacteria such as Escherichia coli (29%) and Klebsiella pneumoniae (16%). Microorganisms with a high degree of resistance to conventional antibiotics were also identified, such as ESBLs (extended spectrum β-lactamases) bacteria. Darkahi B et al. performed analyzes of biliary culture in patients undergoing cholecystectomy for AC and observed bactobilia in 16 (31%) of the 51 cholecystectomy patients. Furthermore, bacterial growth was seen in 18 (9%) of the 195 bile cultures from non-acute cholecystitis, suggesting that bacterial colonization in patients with acute cholecystitis is greater (p<0.001).

Foreign bodies in the biliary tract can facilitate bacterobilia^13^. Yu, J.-L et al. implanted solid objects into the temporarily occluded biliary tract of rats. Electron microscopy showed bacterial colonization and biofilm formation on the surface of the implanted material and on the surface of the biliary tract mucosa^11^. This finding showed that physiological bacterial elimination loses its value when microorganisms attach to a foreign body, such as gallstones. Biofilm formation confers resistance to antimicrobial agents and is responsible for the low positivity of culture tests. Sonication is a method that, through ultrasound waves, promotes the rupture of the polymeric matrix of the biofilm, detaching the sessile bacteria from the surfaces that had adhered without damaging them. The fluid culture resulting from this process seems to be more positive when compared with the conventional culture^12^.

In the present study, we performed analysis of bile and sonication fluid from gallstones taken from patients undergoing cholecystectomy for uncomplicated cholelithiasis and for AC, and analyzed the epidemiological profile resulting from the findings of the gallbladder microbiota and gallstones.

## METHODOLOGY

### Study Design

This is a unicentric, cross-sectional study comparing the results of microbial identification and sensitivity tests of microorganisms identified in bile and sonication fluid from gallstones in patients with cholelithiasis who underwent cholecystectomy for uncomplicated cholelithiasis or for AC in a secondary hospital in the city of São Paulo.

### Study Area

During the period from January 2021 to June 2022, we studied all patients over 14 years of age with cholelithiasis who underwent cholecystectomy for uncomplicated cholelithiasis or for AC at the Hospital São Luiz Gonzaga and Hospital Central da Irmandade da Santa Casa de São Paulo. Gallstones and bile samples were sent to the central hospital of the Irmandade da Santa Casa de Misericórdia de São Paulo. The study was approved by the Ethics in Research Committee (CEP) of the Irmandade da Santa Casa de São Paulo, under number CAAE: 40713420.0.0000.5479.

### Study Population

The population studied consisted of patients treated at Hospital São Luiz Gonzaga and Hospital Central da Irmandade da Santa Casa de Misericórdia de São Paulo, over 14 years of age with cholelithiasis, who underwent cholecystectomy for uncomplicated cholelithiasis or for AC, submitted to a standard surgical procedure, and who agreed to participate in the study by signing the Informed Consent Form. We excluded those with fistulas (Mirizzi syndrome or fistulas to the digestive tract), chronic inflammatory diseases of the biliary tract, biliary malformations, those whose anatomopathological examination of the specimen indicated neoplastic disease, and patients who refused to participate in the study. In addition, we excluded samples in which there was obvious contamination, doubts in identification, insufficient material, or in which the interval between collection and processing in the microbiology laboratory was greater than six hours. We considered a valid case one with successful examination of both the calculus and the bile.

### Study Variables

Regarding the patients and the surgical procedure, we studied age, sex, height, weight, Body Mass Index (BMI), comorbidities (systemic arterial hypertension, diabetes mellitus, chronic renal failure, smoking, chronic obstructive pulmonary disease, alcoholism, chronic hepatitis, cirrhosis, sickle cell anemia, use of corticosteroids, positive serology for HIV), reason for surgery (uncomplicated cholelithiasis or AC), antibiotic therapy in the last two weeks, previous endoscopic manipulation of the biliary tract, access route for removal of the gallbladder (laparotomic, laparoscopic, or laparoscopic converted into laparotomy), type of stone (yellow, brown, or black), and, in cases of AC, classification of severity, C-Reactive Protein (PCR), Alkaline Phosphatase (AP), and Gamma Glutamyl Transferase (GGT).

### Source of Information and Data Collection

All patients underwent cholecystectomy. Bile samples were collected in 0.9% saline solution, bile in thioglycolate and gallstone sonication fluid in 0.9% saline solution. The 102 samples were seeded on blood agar and chocolate agar media in aerobic and anaerobic environments. Bile samples were sent directly to the central laboratory, where they were seeded on blood agar and chocolate agar. The Falcon tubes containing the calculi were sent to the microbiology laboratory of the Faculty of Medical Sciences of Santa Casa de São Paulo, where they were subjected to the following processes:

1. vortexing for 30 seconds using the Vortex-Genie 2 (Scientific Industries, Inc., Bohemia, NY, USA).
2. sonication for 5 minutes at a frequency of 40±2 kHz and density power of 0.22±0.04 W/cm^2^ using the ultrasonic washer model BactoSonic 14.2 (BANDELIN Electronic GmbH & Co. KG, Germany).
3. eddy for an additional 30 seconds.
4. concentration of the sonication fluid through centrifugation performed in 50mL aliquots at 2,500rpm for 5 minutes.

At the end of this process, the resulting fluid was sent to the central laboratory for seeding on blood agar and chocolate agar media (the same ones in which the bile sample was seeded). Bile and sonication fluid samples were stored and frozen at the end of these procedures.

The aerobic plates (blood agar and chocolate agar) were incubated at 35-37°C with 5-7% CO_2_ for 7 days; the anaerobic plates, at 37°C for 14 days. All plates were inspected daily to monitor the growth of microorganisms. Finally, microbial susceptibility tests followed the standards established by the CLSI (Clinical Laboratory Standards Institute) and were performed in all identified colonies. The CLSI standardization for disc diffusion was used across all strains to identify the susceptibility profile. The quality control of the susceptibility test was performed with the standard strains (Pseudomonas aeruginosa ATCC 27853, Escherichia coli ATCC 25922, and Staphylococcus aureus ATCC 25923). The reference standard adopted for these tests is the one used in the laboratory and follows the international criteria of the CLSI.

### Diagnostic Criteria

Uncomplicated cholelithiasis: detection of stones in the gallbladder by any imaging exam (most commonly by abdominal ultrasonography, but those detected by computed axial tomography, magnetic nuclear resonance, or echo-endoscopy would also be accepted) and confirmed after cholecystectomy (anatomopathological exam without acute inflammatory signs).

Acute cholecystitis: detection of stones in the gallbladder by any imaging exam (most commonly by abdominal ultrasonography, but those detected by computed axial tomography, magnetic nuclear resonance, or echo-endoscopy would also be accepted) and confirmed after cholecystectomy (anatomopathological exam with signs acute inflammation).

To classify the severity of acute cholecystitis, we used the Tokyo Guidelines 2018^14^:

- Mild: No complications.
- Moderate: abdominal complications (gallbladder empyema/necrosis, perivesicular abscess, liver abscess, or choleperitoneum), more than 72 hours of pain, or more than 18,000 leukocytes/field on CBC.
- Severe: systemic complications (Glasgow coma scale less than 15, PaO_2_ / FiO_2_ ratio less than 300mmHg, need to use any dose of intravenous noradrenaline to maintain blood pressure, less than 100,000 platelets / µL, INR greater than 1.5, and / or serum creatinine greater than 2mg/dL).

### Surgical Technique

Cholecystectomies were performed either openly or laparoscopically. In the former, the gallbladder was immediately taken to the instrument technician’s table and punctured to extract the bile in a syringe. In the latter, the bile was aspirated through a syringe during the removal of the organ from the abdominal cavity. Patients with an amount of obtained bile less than 5 mL were excluded from the study.

After collection, two bile samples were separated. One of them was placed in a Falcon tube containing 0.9% saline and the other in Thioglycolate. All containers were duly labeled with the patient’s record, date of surgery, and collection time.

### Statistical Analysis

For the sample description, we used percentile and mean for qualitative variables and median and standard deviation for quantitative ones. To compare the results between the different samples (bile and calculus), we used the chi-square, the Fisher, and the Mann-Whitney tests according to the analyzed variables. We considered the value of p<0.05 as statistically relevant. We used the REDCap software to analyze the studied variables.

### Ethical Aspects

This project was initially registered on Plataforma Brasil and forwarded to the Ethics in Research Committee of the Faculty of Medical Sciences of Santa Casa de São Paulo, where it was approved (CAAE: 40713420.0.0000.5479).

Only patients who were informed about the content of the study and who agreed with the terms contained in the Informed Consent Form were accepted in the study.

## RESULTS

### Study Population

During the study period, 34 patients were included. There were no exclusions. Of these, 26 were female (76.4%), 11 were older than 60 years (average 48 +/- 16.61 years, range 23-76), only nine patients were eutrophic (26.4%), while the others were above the ideal Body Mass Index Kg/m^2^ (average 27.64 +/- 4.27, range 15.97-36.44).

### Diseases Found and Surgical Procedures

Upon admission, the 17 patients diagnosed with AC underwent C-Reactive Protein (CRP), Alkaline Phosphatase (AP), and Gamma Glutamyl Transferase (GGT) tests and their respective classification by the Tokyo Guidelines 2018 (TG). The others underwent elective surgeries for uncomplicated cholelithiasis, without the clinical need for the laboratory analysis described in Table 3.

**Table 1:** Demographic analysis of the studied patients and Body Mass Index.

Demographic data	Number of patients (%)
Total	34 (100)
Female	26 (76,4)
Male	8 (23,5)
Age >60 years	11 (32,3)
Body Mass Index (BMI)	
<18.5 (Underweight)	0 (0)
18.5 - 24.9 (Normal weight)	9 (26,4)
25 - 29.9 (Overweight)	16 (47)
30.0 - 34.99 (Grade I obesity)	8 (23,5)
35 - 39.99 (Grade II obesity)	1 (2,9)
>40.0 (Morbid Obesity)	0 (0)

### Surgical Procedure

**Figure 1:**
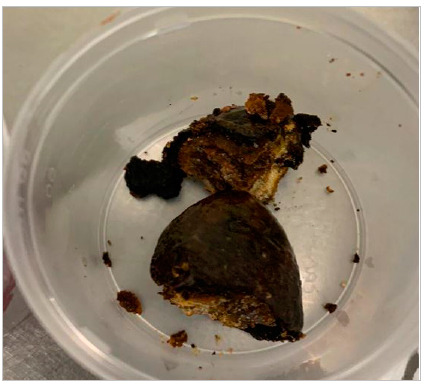
Gallstones present in a patient with Acute Cholecystitis who underwent laparoscopic cholecystectomy.

With the exception of one patient diagnosed with TG III acute cholecystitis, who underwent surgery via laparotomy (right subcostal incision), all patients were operated via laparoscopy (Table 2). Furthermore, there was predominance of yellow-colored calculi (Figure 1).

**Table 2:** Diagnosis and treatment of operated patients.

Diagnosis	Number of patients (%)
Uncomplicated cholelithiasis	17 (50)
Acute cholecystitis	17 (50)
Use of antibiotics	
Antibiotic prophylaxis with Cefazolin	17 (50)
Antibiotic therapy with Ceftriaxone and Metronidazole	17 (50)
Surgical technique	
Laparoscopy	33 (97.1)
Laparotomy	1 (2.9)
Calculi Type	
Yellow	21 (61.7)
Black	9 (26.4)
Brown	4 (11.7)

### Microbiological Findings

There was growth of microorganisms in 32.4% (11/34) of the patients studied (Table 3). In addition, bacteria were identified in 23.5% (4/17) of the patients who received antibiotic prophylaxis with cefazolin and 41.2% (7/17) of those who received antibiotic therapy with ceftriaxone and metronidazole (p=0.271). Regarding the color of the stones, bactobilia was evidenced in 38.1% (8/21) of the yellow stones, in 22.2% (2/11) of black ones, and in 25% (1/4) of brown stones (p=0.658).

**Table 3:** Bacterial growth according to diagnosis.

Condition	Bacterial growth, n (%)		
	No	Yes	p*
Acute cholecystitis	10 (58.8)	7 (41.2)	0.271
Uncomplicated cholelithiasis	13 (76.5)	4 (23.5)	0.463
Total	23 (67.6)	11 (32.4)	0.269

*Chi-square Test.

Table 4 shows the comparative analysis between bacterial growth and the stratification of the clinical picture according to the Tokyo Guidelines 2018 (TG18). We fond no statistical differences between disease severity and pathogen identification.

**Table 4:** Bacterial growth in the acute cholecystitis caes, stratified by the Tokyo Guidelines 2018 sevetiry classification.

Tokyo Guidelines 2018	Bacterial growth, n (%)		
	No	Yes	p*
TG - I	2 (66.7)	1 (33.3)	0.404
TG - II	8 (61.5)	5 (38.5)	0.374
TG - III	0 (0)	1 (100)	0.187

TG: Tokyo Guidelines 2018; I: Mild; II: Moderate; III: Severe; *Chi-Square Test.

There were no statistical differences between the comorbidities studied and bactobilia, as described in Table 6. Table 7 shows the analysis between the quantitative variables in the study (BMI, C-reactive protein, leukocyte count, neutrophils, lymphocytes, Alkaline Phosphatase, and Gamma-glutamyl transferase) and the presence of bacterial growth, using the Mann-Whitney test.

**Table 5:** Comorbidities and the presence of bactobilia.

Comorbidities	n (%)	p*
Systemic Arterial Hypertension	8 (23.5)	0.611
Diabetes Mellitus	8 (23.5)	0.611
Dyslipidemia	2 (5.8)	0.313
Hypothyroidism	3 (8.8)	0.210
Smoking	3 (8.8)	0.210
Chronic Kidney Failure	0 (0)	-
Chronic obstructive pulmonary disease	0 (0)	-
Cirrhosis	0 (0)	-
Hepatitis	0 (0)	-
Sickle cell anemia	0 (0)	-
Other	1 (2.9)	0.142

*Chi-square test

**Table 6:** Quantitative variables and the presence of bacterial growth..

Variable	p *
Body Mass Index (BMI)	0.612
White blood cell count	0.416
Neutrophil count	0.165
Lymphocyte count	0.565
C-Reactive Protein (CRP)	0.094
Alkaline Phosphatase (AP)	0.071
Gamma Glutamyl Transferase (GGT)	0.103

*Mann-Whitney test.

**Figure 2:**
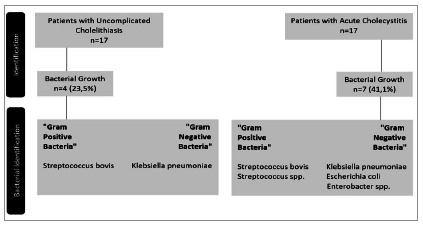
Bacterial growth in acute cholecystitis and uncomplicated cholelithiasis and their respective findings.

**Figure 3:**
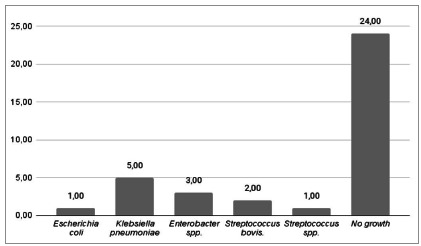
General frequency of microorganisms found.

We observed the highest positivity of microbiological tests in patients with AC (Figure 2). In general, there was an unfavorable sensitivity pattern for the pathogens found, mainly Gram-negative bacteria (Klebsiella pneumoniae and Enterobacter spp.), showing a profile of resistance to first-generation cephalosporins (cefazolin and cephalothin) and to ampicillin. Importantly, these patients did not present a description of antibiotics use before surgery. The isolated Gram-positive bacteria were sensitive to penicillins. Figure 3 shows the relative frequency of the isoated microorganisms, and Figue 4 shows the microorganisms found in each diagnosis.

**Figure 4:**
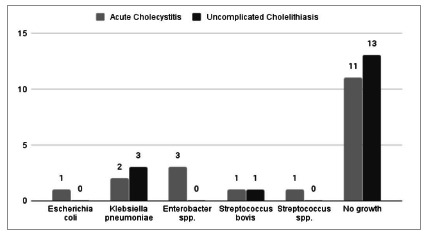
Microorganisms found in Uncomplicated Cholelithiasis and in Acute Cholecystitis.

### Comparison Of Microbiological Findings Between Sonication Fluid From Gallstones And Bile Samples

**Figure 5:**
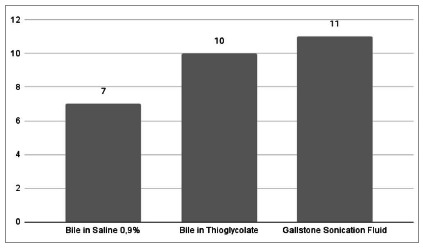
Comparison between samples with bacterial growth.

The sensitivity of identification of pathogens by the sonication test of the calculi occurred in 32.3% (11/34 stones). The sensitivity of identifying bile pathogens was 10.2% (7/68 bile samples) (p=0.0058). Among the cases in which there was bacterial growth, the sample with sonication fluid from the gallstone was positive in all cultures, while in the samples of bile in saline solution 0.9% and in thioglycolate, seven and ten of the cases, respectively (Figure 5). The bile sample in 0.9% saline did not show growth of microorganisms from patients 468681 (Escherichia coli and Enterobacter spp.), 461359 (Streptococcus bovis), 3181108 (Streptococcus spp.), or 6533489 (Klebsiella pneumoniae), while the bile sample in thioglycolate did not show the bactobilia of patient 468681 (Escherichia coli and Enterobacter spp.).

Using the Kappa coefficient, we noticed an important association (Kappa between 0.61 and 0.8) between the methods using bile in saline solution 0.9% and bile in thioglycolate, and between bile in saline solution 0.9% and sonication fluid from the gallstones (Table 7). Furthermore, we observed an almost perfect association (Kappa between 0.81 - 0.99) between bile samples in thioglycolate and gallstone sonication fluid (0.929).

**Table 7:** Agreement index (Kappa) obtained between the collected samples regarding bacterial growth.

Kappa statistics	p
Bile in saline solution 0.9% / Bile in thioglycolate	0.611
Bile in Saline 0.9% / Gallstone Sonication Fluid	0.702
Bile in Thioglycolate / Gallstone Sonication Fluid	0.929

## DISCUSSION

The main finding of this study was the greater sensitivity of pathogen identification when samples of sonication fluid from gallstones were used for bacterial investigation. By amplifying the sampling of microbiological cellular material, this method improves the sensitivity of cultures, in addition to providing sensitivity tests to antibiotics^13^. Such a finding may help us in understanding bacterial colonization in gallstones. In addition to sonication, we also noticed that bile in thioglycolate increased sensitivity when compared with the conventional method.

This work found the presence of bacteria in samples from 41.1% of patients with AC. Other studies suggest the incidence of bactobilia between 35% to 65% of cases of AC and around 10% in those with uncomplicated cholelithiasis^10^ ^,^ ^15^. Despite the statistical limitation, we observed that the prevalence of bacterial growth was higher the more sevre was the patient. It is likely that the bile microflora contained in the gallbladder is important for the clinical-surgical outcome of patients undergoing cholecystectomy^14^. However, further studies are needed to evaluate techniques to prevent postoperative infections and assess their effectiveness.

Despite the lithogenic theory of pigmented calculi, which consists of cholestasis associated with bacterial colonization (Escherichia coli, Klebsiella pneumoniae, and Enterococcus faecalis)^12^, there was a lower prevalence of bactobilia in individuals with brown calculi compared with those with yellow ones. However, such findings were inconclusive and were not statistically relevant in the association between the presence of bacteria in bile or gallstones and the incidence of complications in cholelithiasis. As in previous studies, the enteric bacteria Klebsiella pneumoniae and Enterobacter spp. predominated among positive cultures^10^ ^,^ ^12^ ^,^ ^15^ ^,^ ^16^. It is postulated that such microorganisms reach the gallbladder lumen by ascending the duodenum. Gram positive bacteria such as Streptococcus spp. were also isolated in the study, however it is likely that they also have an enteric origin^14^.

Of the 19 antibiotics used in the sensitivity profiles, ampicillin proved to be inappropriate for the therapeutic management of bactobilia. Furthermore, only 50% (5/10) of the microorganisms were sensitive to cefazolin. R. Reiss et. al. investigated the presence of bactobilia in 800 patients undergoing cholecystectomy. Of these, 27% (217/800) of the cases had bacterial growth and 12% (27/217) were strains of Klebsiella pneumoniae, whose sensitivities to first-generation cephalosporin and ampicillin were 56% and 10%, respectively^19^. In our study, the strains of Klebsiella pneumoniae (5/11) showed 100% resistance to ampicillin and only 20% resistance to cefazolin.

The use of antibiotics in medical practice for the management of AC is widely accepted^19^. Mazeh et al. performed a comparative study between patients with mild AC (TG-I) who used antibiotic therapy and those who did not. They observed that the use of intravenous antibiotics did not affect the length of hospital stay, the number of admissions, or the surgical outcome. Their study suggests that the genesis of AC in these cases is more associated with the inflammatory process than with the infectious process, and that antibiotic therapy should be individualized based on its hospital course^19^. We did not find statistical relevance in the use of antibiotics for prophylactic or therapeutic use in eradicating bactobilia and its clinical outcome in both groups of patients. There is controversy about the use of antibiotics for prophylaxis in cholecystectomy for uncomplicated cholelithiasis^19^. It is postulated that the lack of antimicrobial effect is associated with poor diffusion of drugs in the biliary tract^14^. However, adequate bacterial identification can contribute to decision-making in case of complications, such as intra or postoperative bile leakage^14^.

## CONCLUSION

The prevalence of bacteria found in the bile of patients with acute cholecystitis was higher than that of those with uncomplicated cholelithiasis. However, we found no relevant statistical association between the evaluated clinical and laboratory variables and bacterial growth in the samples. The use of sonication as a method of bacterial investigation proved to be superior to the conventional method of bile culture, and can be considered in the medical routine.
